# Performance Evaluation for Ultra-Lightweight Epoxy-Based Bipolar Plate Production with Cycle Time Reduction of Reactive Molding Process

**DOI:** 10.3390/polym14235226

**Published:** 2022-12-01

**Authors:** Budsaba Karoonsit, Rungsima Yeetsorn, Darunee Aussawasathien, Walaiporn Prissanaroon-Ouajai, Gaurav Kumar Yogesh, Yaowaret Maiket

**Affiliations:** 1 Materials and Production Engineering, The Sirindhorn International Thai-German Graduate School of Engineering, King Mongkut’s University of Technology North Bangkok, Bangkok 10800, Thailand; 2 Advanced Polymer Technology Research Group, National Metal, and Materials Technology Center, Khlong Luang, Pathum Thani 12120, Thailand; 3 Department of Industrial Chemistry, Faculty of Applied Science, King Mongkut’s University of Technology North Bangkok, Bangkok 10800, Thailand; 4 Thai-French Innovation Institute, King Mongkut’s University of Technology North Bangkok, Bangkok 10800, Thailand

**Keywords:** reactive molding cycle time, ultra-lightweight bipolar plate, epoxy composites laminates, carbon nanofillers, degree of crosslinking, reactive molding kinetics

## Abstract

The commercial viability of fuel cells for vehicle application has been examined in the context of lightweight material options, as well as in combination with improvements in fuel cell powertrain. Investigation into ultra-lightweight bipolar plates (BPs), the main component in terms of the weight effect, is of great importance to enhance energy efficiency. This research aims to fabricate a layered carbon fiber/epoxy composite structure for BPs. Two types of carbon fillers (COOH-MWCNT and COOH-GNP) reinforced with woven carbon fiber sheets (WCFS) have been utilized. The conceptual idea is to reduce molding cycle time by improving the structural, electrical, and mechanical properties of BPs. Reducing the reactive molding cycle time is required for commercial production possibility. The desired crosslink density of 97%, observed at reactive molding time, was reduced by 83% at 140 °C processing temperature. The as-fabricated BPs demonstrate excellent electrical conductivity and mechanical strength that achieved the DOE standard. Under actual fuel cell operation, the as-fabricated BPs show superior performance to commercial furan-based composite BPs in terms of the cell potential and maximum power. This research demonstrates the practical and straightforward way to produce high-performance and reliable BPs with a rapid production rate for actual PEMFC utilization.

## 1. Introduction

Proton exchange membrane fuel cells (PEMFCs) have received attention for evolution from laboratory and pilot scales to commercialization because of their inherent benefit of electrochemical conversion with high conversion efficiency. Specific attention has been paid to light-duty vehicles since they provide fast charging and long-range [1]. According to demands for real commercialization, considerations should cover issues related to a lightweight design and structural optimization [2]. A current investigation indicates that the effectiveness of weight reduction in enhancing fuel economy is diminished in hybrid or electric vehicles compared to internal combustion vehicles [3]. This is principally due to their capability of apprehending some of the recaptured energy, thus; decreasing the weight-related fuel consumption influences on braking and idling [4]. Even if PEMFCs possess a high-energy density, PEMFCs are typically too heavy, rigid, and bulky to be used in light-duty fuel cell electric vehicles such as scooters, bicycles, city cars, and drones [5,6,7,8,9]. An adequate fuel cell powertrain for the given applications depends upon weight and volume limitations, vehicle power, energy requirements, and the characteristics of the fuel cell stack [8]. To achieve the demands of mobility applications, researchers have attempted to optimize the design and fabrication of the key components, in the weight effect, such as bipolar plates, current collectors, end plates, and stack hardware [10,11]. The increase in hydrogen storage density, storage techniques, and the development of lightweight hydrogen cylinders was also addressed in order to reduce the weight of fuel cell propulsion systems for light-duty vehicles [12]. Thus, light-weighting methods and material designs are expected to be more viable in light-duty fuel cell electric vehicles than in conventional vehicles. A bipolar plate (BP) is one of the most important components of PEMFC that contributes considerably to the weight of the PEMFC stacks and the cost structure. BPs account for nearly 70% of the PEMFC weight and 30–45% of the cost of PEMFCs [10,11]. Lower weight or fewer bipolar plates is demanded to acquire a generated output power with the desired electrical transport between fuel cells in a stack that leads toward market acceptance. This information inspired the development of ultra-lightweight BPs for lightweight fuel cells. Using efficient materials and suitable manufacturing processes is directly proportional to the production cost and required properties. The desired properties are related to the functions of BPs which are electrically connecting the cells in a series, distributing the reactant, preventing the water/gas leakage, separating the gases in the adjacent cells, and even transferring the heat out of the cells [13,14]. Typically, BPs are made from graphite, a lightweight, corrosion-resistant, and electrically conductive material. Nevertheless, it is quite expensive, brittle, and difficult to machine. Hence, very thick graphite (several millimeters) is required for BP fabrication, resulting in a massive and voluminous fuel stack [15]. A polymer/conductive filler composite with either thermoplastic or thermoset matrix is a good material choice for a BP fabrication to its low weight, and it can be produced in an economical process such as compression molding, transfer compression molding, and injection molding. There are several different polymer types of commercially available polymer composite BPs such as polypropylene (PP), polyphenylene sulfide (PPS), polyvinylidene fluoride (PVDF), fluoropolymer, polyphenylene sulfide (PPS), phenolic resin, and epoxy [16,17]. Because of the electrical conductivity requirement, the pathway to tailor advanced materials is to incorporate conductive fillers such as high conductive carbon black, graphite (exfoliated graphite, expanded graphite, or synthetic graphite), multi-walled and single-walled carbon nanotubes, carbon fibers, and graphene, in the polymer matrix [18]. The polymer composite’s conductive network and the mechanical strength of the polymer composite are relevant to the filler loading, morphology, processing, and size of the added particle [19]. Epoxy composites are an attractive option for commercial BP production since they offer several advantages, including low cost, lower weight, and greater ease of manufacture. Compared with conventional graphite or metals, epoxy composite has high specific strength and stiffness [17,20]. The properties of the epoxy composite can be tailored to reinforcement types, filler systems, and filler loading [21,22]. In terms of the reinforcement types, carbon fiber-reinforced polymer composites, carbon-carbon composites, hybrid composites, and structural composites have been created for the improvement of mechanical strength and electrical conductivity [23,24]. The structure composites can be divided into two categories which are sandwich panels and laminated composites [25,26]. In this work, the laminated composite was selected to produce an electrically conductive BP. For high-performance PEMFC production, the DoE Standard established the goal for electrical conductivity > 100 S/cm, specific aerial resistance < 0.01 Ωcm^2^, and flexural strength > 59 MPa for BPs [27]. In terms of structure composites, multiwalled carbon nanotubes (MWCNTs) and graphene nanoplatelets (GNPs) in carbon fiber reinforced polymer (CFRP) composites have received attention for improving interlaminar mechanical and electrical properties of composite laminates [26]. The MWCNTs with a one-dimensional (1-D) cylindrical shape and GNP with a two- dimensional (2-D) shape and multi-graphene layers possess hexagonal structures of sp^2^ hybridized carbon atoms [22]. Their characteristics are not only useful for stress transfer from the epoxy matrix, but they are also beneficial to the electrically conductive network formation. D. Aussawasathien, who is one of the co-authors, studied the application of the nano-structure approach of mixing nanofillers of carboxylic-plasma-functionalized graphene nanoplatelet (COOH-GNP) and carboxylic-plasma-functionalized multiwalled carbon nanotubes (COOH-MWCNT) to improve the interlaminar interface of epoxy/carbon fiber composite laminates [28]. This nano-structure establishes a short transport pathway between the carbon fiber of the adjacent laminates. The maximum electrical conductivity and the highest flexural strength of this structure composite were approximately 75 S/cm and 675 MPa. This work brought about the perspective to utilize this epoxy composite as a BP material. Nevertheless, the epoxy-CF composite laminate was fabricated via compression molding for 60 min. This cycle time is quite long and does not achieve commercial production acceptance. There is a necessity to precisely monitor and control the curing process to improve the processability and quality of BP composites. Even though an adequate cure time is established to assure that the cure reaction will be completely finished, conservative estimates may increase the manufacturing cycle times resulting in over-curing. The over-manufacturing cycle time and over-curing lead to high capital costs and may bring about poor properties of the BP product. There is a need to be mindful of the difficulties of molding BPs since the BPs comprise complicated gas flow channels. The ideal molding and demolding conditions are crucial for producing high-quality BPs with intricate gas flow channels. Modeling the kinetics of material reactions gives researchers necessary information for the process development and prediction of optimum reaction temperatures, process control by optimization of reaction advancement sor conversion, and an estimation of material lifespan [29]. Numerous analytical techniques, including differential scanning calorimetry, dynamic mechanical analysis, dielectric analysis, Raman spectroscopy, infrared spectroscopy, ultrasonic method, and rheological characterization have been used to evaluate the cure reaction kinetics and monitor the curing process of thermoset [29,30,31]. An effective path to explore the process from the kinetics matter is using DSC that can be used to evaluate kinetic parameters such as reaction activation energy and the order of physic-chemical processes in highly exothermic reactions such as crosslinking [31]. DSC has been used to investigate the reactions between polymer chains or between inorganic fillers, while the study about interaction in polymer/carbon filler composite has not been reported extensively [32]. DSC was used to predict or determine the kinetic of epoxy polymerization and degree of crosslink based on the Arrhenius Equation, and there are several models that have been applied for this investigation. The Kissinger’s model [31,33,34,35,36] was basically used for dynamic conversion, while the Flynn–Wall–Ozawa model [31,33,34] was applied for iso-conversion. The Vyazovkin model [36,37,38] and Coats-Redfem model [39] were employed to predict the polymerization kinetics of the epoxy.

The goal of this research is to better understand and determine the kinetic parameters that can be used to mold materials in a way that will influence the controlling and curing processes during compression molding. The cure behavior was characterized using differential scanning calorimetry (DSC) as a baseline comparison, and the best-fit phenomenological reaction model was conducted to explain the cure behavior of the epoxy composites and the kinetic factors. The obtained results were used to impose the criterion for optimizing the reactive molding conditions to reduce the cycle time of the reactive molding. The structure composites created from this experimental activity are the combination of epoxy, woven carbon fiber sheets (WCFS), carboxylic-plasma-functionalized graphene nanoplatelet (COOH-GNP), and carboxylic-plasma-functionalized multiwalled carbon nanotubes (COOH-MWCNT). The final target of the composite manufacturing corresponds to the required degree of crosslink and filler dispersion, and those characteristics impact the final properties of BPs, which are surface electrical conductivity, volume electrical conductivity, and flexural strength regarding the DOE standard. The created BPs were assembled into a single fuel cell that was operated under actual fuel cell operation to observe reliability for the fuel cell application. 

## 2. Materials and Methods

### 2.1. Materials

Epoxy resin (Modified Bisphenol A diglycidyl ether) and curing agent (Modified Isophorone diamine) supplied from Aditya Birla Chemicals Ltd., Rayong, Thailand were utilized as the main reactants for the curing process. The conductive fillers used to form an electrically conductive network include carboxylic-functionalized graphene nanoplatelets (COOH-GNP), multi-walled nanotubes (COOH-MWCNT), and woven carbon fiber sheet (WCFS). COOH-GNP and COOH-MWCNT were purchased from Haydale Technologies Co., Ltd., Pathum Thani, Thailand. The COOH-GNP have a basal planar length of 0.3–5.0 µm, a thickness < 50 mm, a density of 2.15 g/cm^3,^ and a specific area ≅ 20 g/m^2^. The COOH-MWCNT possess a nanotube diameter of ≅ 9.5 nm, a tube length of ≅ 1500 nm, a density of 1.30 g/cm^3^, and a specific area of 250–300 m^2^/g. Woven carbon fiber sheet (WCFS, Mitsubishi Rayon Co., Tokyo, Japan) contains a filament count of 3000 and a yield of 200 g/m^2^ [28].

### 2.2. Bipolar Plate Fabrication

The epoxy resin: curing agent ratio at 70:30%wt was used throughout the fabrication step. Noted, this ratio is the best formula obtained from the previous experiments that were performed by D. Aussawasathien et al. [28]. The COOH-GNP: COOH-MWCNT ratios (100:0, 50:50, and 0:100%wt) were varied to prepare epoxy/nanofiller suspensions with a constant nanofiller content of 4 phr. To prepare nanofiller suspension with good uniformity, nanofillers were dispersed in epoxy resin using ultra-sonication (DTH Digital control, Branson Ultrasonic, Danbury, CT, USA) at 25 °C of atmospheric temperature for 1 h, and then the suspension was stirred by a high-speed mixer (RW 20 digital, IKA Works GmbH & Co., Staufen, Germany) with a rotational speed of 1000 rpm for 30 min. The curing agent was continuously introduced into the suspension prior to stirring it in the high-speed mixer with the same rotational speed for 5 min. Before a hand-lay-up process, the WCFS surface was cleaned with ethanol to eliminate organic compounds and impurities, and it was subsequently dried in an oven to evaporate ethanol from the WCFS surface. WCFS typically contains polymer binder, so the polymer-rich layer on the WCFS surface can increase contact resistance in the composite laminate disrupting the conductive network creation. The dried WCFS surface was treated by flame burning to alleviate this problem. Five WCFSs with a dimension of 60.0 mm × 60.0 mm × 0.3 mm were laid up one by one layer as a hand-lay-up process and the epoxy/nanofiller mixture was applied on each WCFS layer as a binder. The stack of the composite was placed in the mold designed with gas flow channels. The flow channel configuration is called a three-serpentine pattern. The composite plaque was completely laminated in a compression molding machine (Lab-tech Engineering Co., Ltd., Samut Prakan, Thailand) at 140 °C, under a pressure of 1500 psi. The different curing time was observed to optimize the molding cycle time. The final BP has the dimension of 60.0 mm × 60.0 mm × 1.0 cm with 25 cm^2^ of the flow channel area. 

### 2.3. Characterizations

#### 2.3.1. Curing Kinetic Analysis

The investigation using differential scanning calorimetry (Mettler Toledo DSC 3+) can be separated into two parts; finding the optimum temperature and time for the curing process, and studying the effects of conductive nanofillers on the curing reaction efficiency. The first part is relevant to enthalpy determination associated with dynamic test mode, under a constant nitrogen flow of 50 mL/min, when the sample was heated from −5 to 275 °C. In this scenario, heating rates were varied as 2.5, 5.0, 10.0, 20.0, and 40.0 °C/min, and the samples were a pure epoxy and an epoxy/COOH-GNP/COOH-MWCNT composite with 50:50 of COOH-GNP/COOH-MWCNT ratio. In the second part, the laminated composites with one and two nanofiller systems were selected for measuring heat flow via a dynamic testing technique. The measurement was run by heat-cool-heat step, under a constant nitrogen flow of 50 mL/min, with a constant heating rate of 10.0 °C/min and the temperature sweep was in the range of −5 to 275 °C. 

The obtained heat flow has been applied for the calculation of conversion which was the imperative factor for the Arrhenius kinetic model as explained in Equation (1) [40]. The model was used to compute the activation energy of the reaction related to the optimum temperature and time for controlling the degree of crosslink. The reaction rate, defined as the change in conversion per unit time, is a function of the conversion α.

(1) $$\frac{d\alpha }{\mathrm{dt}}=\mathrm{kf}\;\left(\alpha \right)$$

where α is the degree of cure, t is the reaction time, k is the time-dependent reaction rate constant, and f(α) is some function of the degree of cure. The rate constant is usually considered to have an Arrhenius-type temperature dependence. 

The temperature dependence of the reaction rate constant, k, is described by the Arrhenius Equation (2). Substituting k by Arrhenius equation in the rate equation gives the following Equation (3).

(2) $$K\;\left(T\right)={\;K}_{0}{\;\exp}^{-E\alpha /\mathrm{RT}}$$

(3) $$\frac{d\alpha }{\mathrm{dt}}=K_{0}{\;\exp}^{-E\alpha /\mathrm{RT}}$$

where K (T) is the rate constant at temperature, K_0_ is the rate constant at infinite temperature (pre-exponential factor), g(α) is the reaction model, E is the activation energy, and R is the universal gas constant (8.314 J/mol K). Insertion of Arrhenius equation and the definition of β = dT/dt to Vyazovkin model (Equation (4)) and the assumed to be independent of β give the following Equation (5) [36,38]. Moreover, it can also provide information about thermal transitions, such as the glass transition temperature (Tg).

(4) $$\frac{d\alpha }{\mathrm{dt}}=\frac{K_{0}}{\beta }\exp\left(\frac{-E_{a}}{R}\right)f\left(\alpha \right)$$

(5) $$g\left(\alpha \right)=\frac{K_{0}}{\beta_{1}\;}\;\left(E_{\alpha ,}T_{\alpha ,\beta_{1}}\right)=\frac{K_{0}}{\beta_{n}\;}\;\left(E_{\alpha ,}T_{\alpha ,\beta_{n}}\right)$$

Changes in chemical bonding that occur after the curing reaction has ended were investigated using spectroscopy characterization including Attenuated total reflectance Fourier Transforms Infrared Spectroscopy (ATR-FTIR, Bruker Invenio S, Germany) was recorded in the range of 4000–400 cm^−^^1^ with a spectral resolution of 4 cm^−^^1^ for 32 scans and X-ray photoelectron spectroscopy (XPS) using a Kratos Axis Ultra DLD and monochromatic Al K radiation (Emission 10 mA Anode HT 15 kV) at an operating power of 150 W.

#### 2.3.2. Electrical Conductivity and Mechanical Property Measurements

The surface and volume conductivity of the laminated composite BPs with 100 mm × 100 mm × 1 mm (width × length × thickness) of specimen dimension. The surface conductivity was determined using a four-point probe (SP4) with a source meter (2400C), according to ISO 3915, while the volume conductivity was tested under 0.974 MPa of compressive force with DC power supply (PR18-5A, KENWOOD, Tokyo, Japan) and a nanovolt meter (2182A, Keithley, Solon, OH, USA). In the case of volume conductivity, the test was set following the testing method for bipolar plates [41]. The laminated composite plaque was located between two gas diffusion layers, and all layers were inserted between the gold-coated copper plate. 

The mechanical behavior observation was divided into two parts including static and dynamic modes. Flexural strength and compressive strength were measured in the case of the static mode. The flexural strength was measured with a pressing rate of 0.01 mm/min by a three-point bending test using a Universal tensile machine following ASTMD790, and the specimen dimension was 10.0 mm × 50.0 mm × 1.0 mm (width × length × thickness). The compressive strength was tested according to ASTM D695 with a compressive rate of 1 mm/min at 10% deformation, and the specimen dimension was 12.7 mm × 12.7 mm × 1.0 mm. In terms of the dynamic mode, a cyclic compression test was carried out under 0–0.974 MPa of compressive force, 1 mm/min, and 1000 cycles of repetition.

#### 2.3.3. Bipolar Plate Performance Diagnosis

An in-house single-cell PEMFC acquiring 25 cm^2^ of an active area was installed using a commercial membrane electrode assembly including the Nafion 212 membrane with 0.40 mg/cm^2^ (40% catalyst loading) of Platinum catalyst at both anode and cathode sides, gas diffusion layer (SGL carbon paper). The test station utilized throughout this research activity was developed by researchers at King Mongkut’s University of Technology North Bangkok [42]. During the operation, the hydrogen gas (42 mL/min or 1 bar of pressure) and air (70 mL/min or 2 bar of pressure) were fed to the anode and cathode at 80 °C with 100% relative humidity. The single-cell performance was evaluated by measuring the polarization characteristics using a Potentiostat (Vionic, Metrohm). The maximum power is 2.20 W at 5.50 A and 0.40 V.

## 3. Results and Discussion

### 3.1. Predicting Reaction Behavior from DSC to Minimize Cycle Time for BP Molding

Various techniques have successfully produced lightweight composite materials, but the most practical process is compression molding. Much attention has been paid to material selection and design, while few have focused on optimizing the molding process parameters of manufacturing BP with various gas flow channel configurations. The previous study has found that temperature, time, and compression pressure are crucial factors when shaping via compression molding—according to an industrial attitude, optimizing these factors to achieve the maximum process ability and maintain the desired quality of BPs. Reducing the molding cycle time is vital because that can contribute directly to higher productivity and lower production costs. It is essential to ensure that the excessive cycle time reductions will not cause defect risks in BP specimens. 

**Figure 1:**
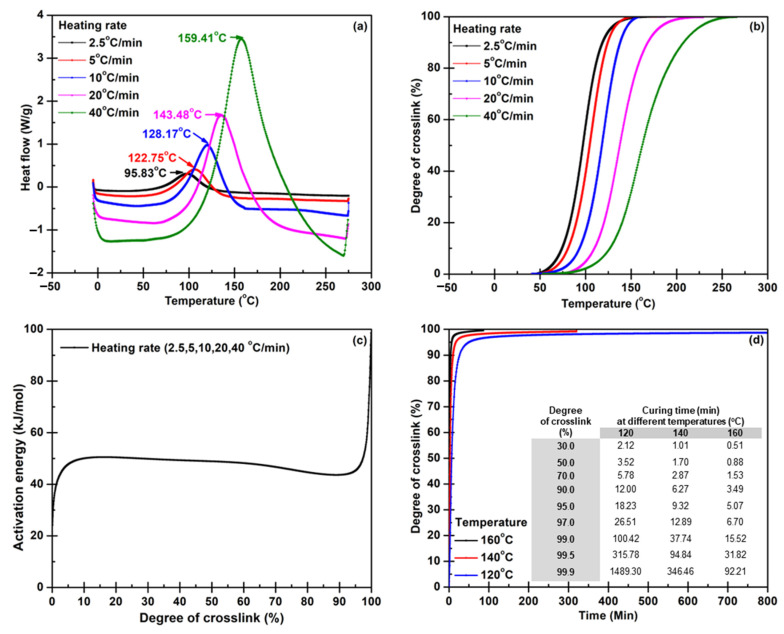
Data: (**a**) heat flow, (**b**) conversion, (**c**) activation energy, and (**d**) optimum temperature and time, obtained from the model-free kinetic calculation for a pure epoxy system.

In the previous report [28], it took about 60 min to prepare an epoxy with nanofiller hybrid composite laminates. Thus, this experimental section focused on studying reaction kinetics by thermal analysis, DSC. Thermal analysis kinetics is a method for investigating and predicting reaction behavior under different conditions. The kinetics can provide information about the influence of temperature and time on curing behavior. The DSC thermograms of the pure epoxy measured at five different heating rates (from 2.5 to 40 °C/min) are shown in Figure 1a. It can be observed that the exothermic peaks (at 95.83, 122.75, 128.17, 143.48, and 159.41 °C) shifted slightly to a higher temperature when the heating rate was increased since the rapid reaction between the epoxy resin and the curing agent was generated. Enthalpy values from those DSC thermograms were used to calculate conversion in the next step. The conversions of each reaction measurement illustrated the degree of crosslink at different heating rates (Figure 1b). The calculation results indicated that the initial reaction and end temperatures shifted to a higher temperature when the heating rate was elevated. At the same time, the slopes of the curing percentage curves insignificantly differed at a high curing percentage. The data from the conversion calculation part were applied to determine the activation energy (Ea) under different heating rates via Model Free Kinetic (MFK) following Equation (5). Ea increased from 20 to 50 kJ/mol with the increase in % curing from 0 to 10%; the Ea slightly rose from 50 to 55 kJ/mol as % curing rose from 10 to 20%. When the % curing escalated from 20 to 90%, Ea values decreased from 50 to 45 kJ/mol. The Ea rapidly increased from 45 to 100 kJ/mol if the % curing scaled from 20 to 90%. These changes presented phenomenological behavior due to crosslinking generation in the thermosetting matrix, resulting in highly restricted molecular mobility [43]. The curing temperature and time, illustrated in Figure 1d, were optimized using the computed activation energy. 

**Figure 2:**
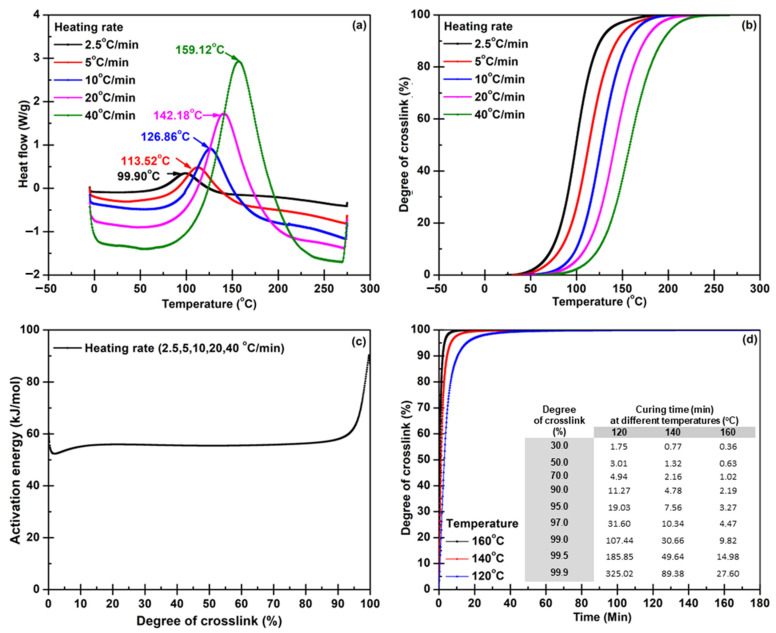
Data: (**a**) heat flow, (**b**) conversion, (**c**) activation energy, and (**d**) optimum temperature and time, obtained from the model-free kinetic calculation for epoxy/nanofiller hybrid composite.

Since the lightweight BPs have to be electrically conductive, the electrical nanofillers were involved in the composite system. To determine the optimum condition for curing epoxy composite, the effects of the nanofillers on the curing factors were concerned. Figure 2a illustrates the dynamic DSC thermograms for the composite epoxy resin system measured in the same condition as the pure epoxy. The exothermic peaks show the same shifting trends as the epoxy system did. The exothermic peaks measured with heating rates of 2.5, 5, 10, 20, and 40 °C/min appeared at 99.90, 113.52, 126.86, 142.18, and 159.12 °C, respectively. The calculation results of % curing revealed that the reaction and temperatures shifted towards higher temperatures with the heating rate increasing (Figure 2b). It was observed that the conversion at higher heating rates of 20 and 40 °C/min progresses fast at low heating rates since the reaction transformed from a kinetic reaction to a diffusion-based one. The f(α) model function was used to determine the curing reaction following Equation (1), and the f(α) can be calculated from the quotient of each reaction conversion (dα/dt) divided by rate constant at certain temperatures. The conversion value indicates the degree of crosslink, and the degree of crosslink can be determined as a function of time. The rate constant can be experimentally determined by varying reaction temperatures. The activation energy values with different heating rates calculated by Model Free Kinetic (MFK) are illustrated in Figure 2c and the suggested conditions corresponding to curtained degrees of crosslinks were exhibited in Figure 2d. Interestingly, the curing times of the composite for various reaction temperatures were much shorter than the curing times of pure epoxy. It is because the nanofillers acquire high thermal conductivity [44], therefore; well heat distribution in the composite system promotes the curing kinetics. Thus, the curing rate dramatically increases. Another doubt is relevant to the reaction generated on surfaces of functionalized nanofillers in the composite system. This issue was proven, and the results are reported in the next section. The optimum molding time is determined by taking the results from the kinetic study into account with the degree of crosslink that leads to mechanical properties that must meet the intended BP application. The suitable time is reported in mechanical property results.

In conclusion, for this part, 140 °C is a suitable temperature for BP composite molding because of temperature limitations for curing agent use and the easiness of demolding. The specification of modified Isophorone diamine suggests the reaction temperature in the range of 120–150 °C, then the temperature will cause chemical degradation [40]. In an actual reactive molding process via a compression molding machine, the time spent to remove the BP specimen from the mold is 30 s after the curing reaction is terminated. The critical factor is the demolding temperature which should be 120 °C for keeping desired BP dimension and configuration. Even a high operating temperature of 160 °C offers faster curing time, but it will take around 2 min for decreasing mold temperature to the imposed demolding temperature. As a result of rational restriction, 140 °C was selected for the reactive molding process. 

### 3.2. Reactions Generated in the Laminated Composite during the Curing Process

**Figure 3:**
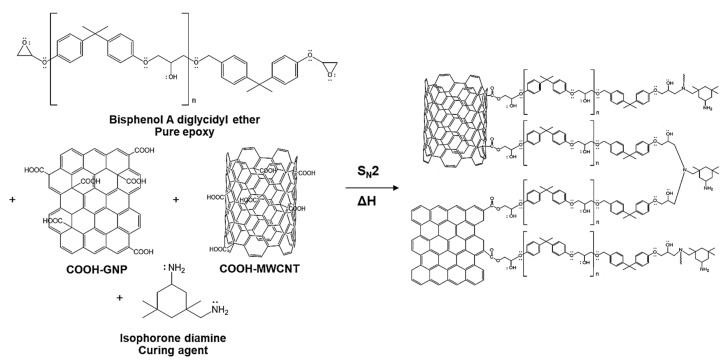
Proposed chemical reactions occur during a reactive molding process.

As the aforementioned matter related to the effect of functionalized nanofillers on curing time reduction, the changes in existing functionalities on nanofiller surfaces and epoxy chains were investigated using FT-IR and XPS. The research literature presented the reaction mechanisms for COOH- Carbonous fillers [30,45,46,47,48] and the mechanism illustrated in Figure 3 are the reaction pathways that can be generated in our laminated composite system. 

**Figure 4:**
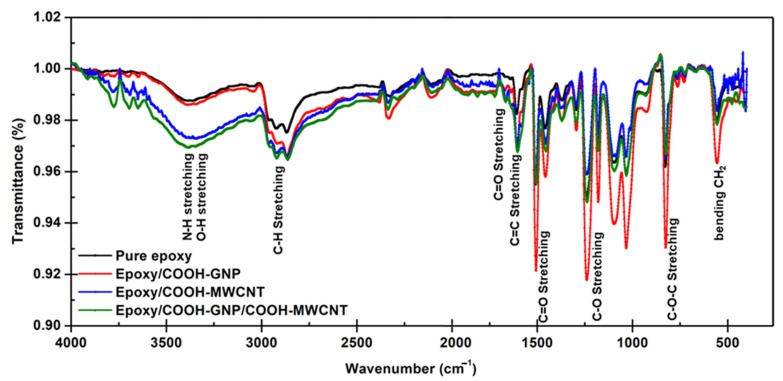
IR results of pure epoxy and epoxy composites with different nanofillers.

Figure 4 shows FT-IR spectra of pure epoxy and epoxy composites with different nanofillers. All FT-IR spectra exhibit distinct absorptions of the oxirane ring including C-O-C stretching (830 cm^−1^), C-O stretching (1250 cm^−1^), and C-H stretching (3000 cm^−1^). Moreover, C=C stretching (1650 cm^−1^), C=O stretching (1505 cm^−1^) of aromatic rings, C-H stretching (2850 cm^−1^), and C-H bending (550 cm^−1^) attributed to the C-H tension of the methylene group of the epoxy ring and CH aromatic and aliphatic are observed for all FT-IR spectra [49,50]. More pronounced peaks at 1720 cm^−1^ assigned to C=O in carboxylic groups are observed only for the epoxy composites confirming the incorporation of carboxylic-plasma-functionalized nanofillers. However, FT-IR results are limited to clarify the chemical bonding between the COOH-MWCNT and COOH-GNP with epoxy chains.

**Figure 5:**
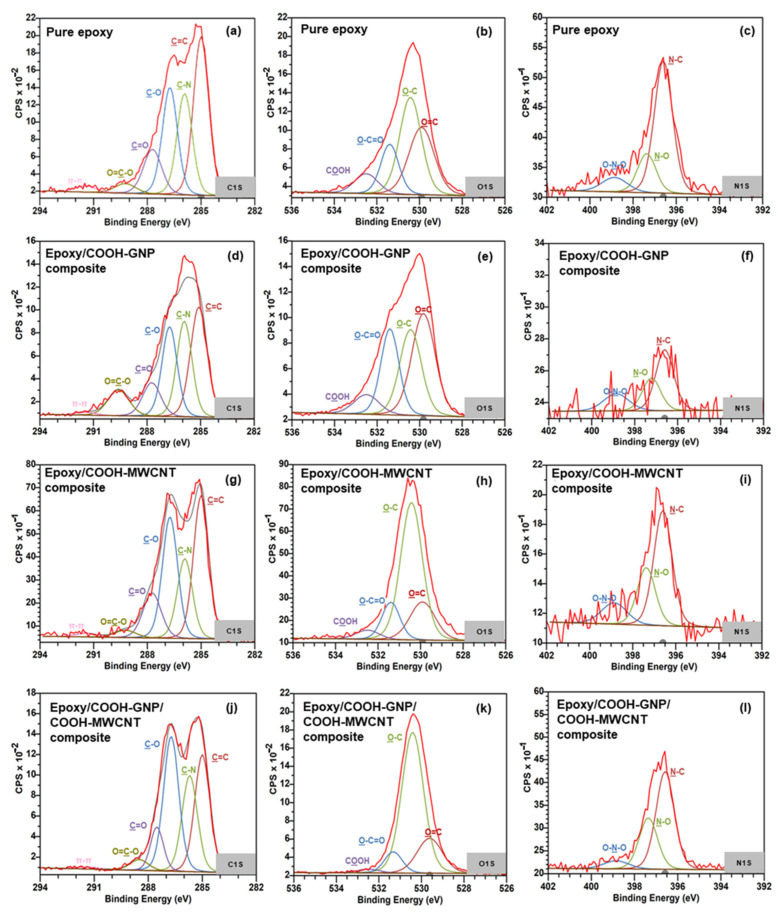
High resolution of XPS spectra of (**a**–**c**) Pure epoxy, (**d**–**f**) Epoxy with COOH-MWCNT, (**g**–**i**) Epoxy with COOH-GNP, and (**j**–**l**) Epoxy with COOH-MWCNT and COOH-GNP.

XPS is a surface science technique used to reveal the elemental composition and chemical bonding of the elements within a material. XPS has been performed here to study the chemical interaction generated in the laminated composite system. As shown in Figure 5a–c, it can be observed that the pure epoxy is divided into three elements, including C1s, O1s, and N1s. The first element contains deconvoluted C1s data and the assigned carbon bonding. The C1s peak is deconvoluted into seven components, which are related to 35.81% of C=C (285.00 eV), 22.87% of C–N (285.92 eV) from amine, 24.18% of C-O (286.74 eV), 12.13% of C=O (287.71 eV), from hydroxyl and epoxide groups, and 3.98% of O=C–O from carboxyl (289.23 eV), as well as 1.94% of two π→π* transitions (291.44 eV [51,52,53,54,55]. The second spectra contain deconvoluted O1s data and the assigned carbon bonding. The O1s peak is described in terms of three contributions which are related to 32.92% of C–O (530.43 eV), 49.68% of C=O (529.91 eV), from hydroxyl and epoxide groups, and 17.37% of O=C–O (531.40 eV), and 9.03% of COOH (532.50 eV), from carboxyl. The last one contains deconvoluted N1s data and the assigned carbon bonding. The N1s peak is described in terms of two contributions which are related to 68.92% of C-N (396.60 eV), 20.44% of N-O (397.37 eV), and 10.64% of O-N-O (398.86 eV), which are from the epoxide group crosslinked with the amine curing agent. The previous research discussed a theoretical study on the epoxide group crosslinked with the amine curing agent, the epoxy groups may be opened by acid occurring via a nucleophilic attack of the amine nitrogen on the terminal carbon of the epoxy [45,46,47]. The plasma functionalization on nanofiller surfaces was found to be an effective means to enhance the interaction between polymers and plasma-modified nanofillers owing to both physical (surface roughness) and chemical treatment (active polar groups) [30,56]. The high-resolution XPS spectra of plasma functionalization on nanofiller surfaces, the C1s of COOH-MWCNT and COOH-GNP are 6.01, 4.73% of O-C=O (289.23 eV), respectively. Meanwhile, the O1s of COOH-MWCNT and COOH-GNP are 31.18, 34.14% of O=C-O (531.40 eV) and 15.96, 25.26% of COOH (532.50 eV), respectively. It could be attributed to the COOH-groups on nanofiller surfaces, resulting in solid interfacial interaction between COOH-nanofillers and the epoxy resin [30,48]. 

The XPS spectra of epoxy with COOH-GNP, the C1s of C-O, C=O, and O-C=O peak is the renamable pure epoxy in the case of adding COOH-GNP as illustrated in Figure 5d–f. This is due to the hindering of COOH-GNP resulting in a chemical reaction between the epoxy resin and curing agent, anyhow; a reaction between some COOH-GNP and epoxide is partially generated. It can be confirmed by the low signal of the O1s in the O-C=O and COOH peaks and N1s in the C-N peak. Figure 5g–i illustrates that the C1s of C-O, C=O, and O-C=O peaks are higher than those of pure epoxy since GNPs act as an acid catalyst resulting in an increase in kinetics rate. Moreover, the reaction between GNPs and epoxy groups may compete against the reaction between epoxy and amine groups [34,57,58,59,60]. In terms of the epoxy composite including COOH-MWCNT and COOH-GNP with a 1:1 filler ratio, the COOH-MWCNT react with oxirane ring as found from C1s appearing at the C-O, C=O, and O=C-O peaks. This phenomenon occurs in the single filler system Figure 5j–l. Table 1 presents Gaussian fitting parameters for XPS spectra interpretation. Experimental results make it clear that COOH-MWCNT and COOH-GNP can form covalent bonds with epoxy chains advocating well filler dispersion and electrically conductive network formation [21]. Moreover, the atomic attraction between filler particles in a polymer matrix enhances the mechanical strength of the composites, especially stiffness and toughness [20]. 

**Figure 6:**
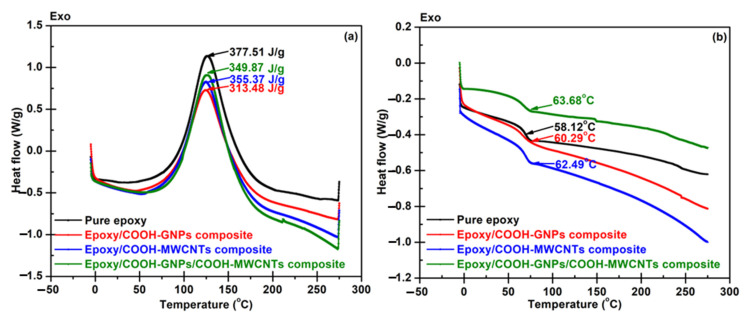
DSC results of pure epoxy and epoxy composites with different nanofillers: (**a**) heat flow, and (**b**) glass transition temperature.

Figure 6a presents exothermic-DSC thermograms of pure epoxy and epoxy composite for observing crosslinking behavior. Theoretically, if the epoxy or composite possesses higher crosslink density, the measured enthalpy of the curing reaction decreases due to the high thermal energy release [61]. The enthalpy was determined by integrating the area under the thermogram peaks. The results show that epoxy composites provided higher crosslink density. It implies that the overall presented enthalpy consists of the releasing energy from reactions between fillers and epoxy and crosslinking between epoxy molecules. All composites have exothermal heat flow less than pure epoxy, possibly due to the crosslinks and bonds on the filler surfaces. From the results, the COOH-GNP seems to have more chance to react with epoxy chains, possibly because of high surface areas which are highly active. In the point of glass transition temperature (Tg) (Figure 6b), the higher Tg indicates the glassy state of materials as well as higher stiffness [38]. In this scenario, the Tg cannot explain an increase in the degree of crosslink clearly, since there is the retardation of polymer chain movement and rearrangement. Filler particles confine the movement of epoxy chains leading to higher energy requirements for the polymer state transition from a glassy state to a rubbery state. The COOH-MWCNT seems to have more impact on Tg than the COOH-GNP does, while the filler hybrid system delivers the highest hindrance effect. 

### 3.3. Relation between Cycle Time Consideration and Mechanical Property Requirement

**Figure 7:**
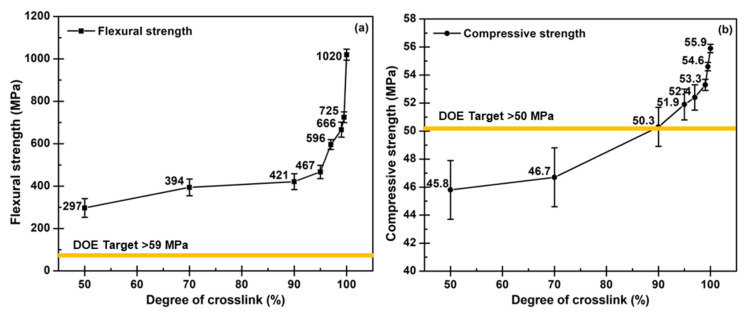
The relation of mechanical strength: (**a**) flexural strength, and (**b**) compressive strength, of laminated composites and different degrees of crosslinks.

As known, materials used to construct BPs must be formable to a structure and have proper properties for machinery operation to make channels in various patterns. Moreover, the fuel cells are typically assembled with compressive force, to protect against gas leakage and reduce the contact resistance of each component [19]. Before curing, epoxy resins have active epoxide or oxirane groups at the ends of the molecules and a repeating unit in the middle of the molecules [62]. They can be any compounds having one or more epoxy groups that can chemically convert to thermosetting materials. Through the ring-opening reaction, the active epoxide groups in the uncured epoxy can react with curing agents containing hydroxyl, carboxyl, amine, or amino groups [38,45,51]. Epoxy resins are produced to offer excellent chemical resistance, excellent adhesion, good heat and electrical resistance, low shrinkage, and good mechanical properties, such as high strength and stiffness. Nevertheless, epoxy resins possess low toughness after curing and are very inclined to inferior resistance to mechanical shocks and vibrations [63]. The properties after curing are not suitable for the bipolar plate application. Therefore, epoxy resin was selected as a matrix for conductive polymer composite BPs in this work. Reinforcing fillers acquiring electrical conductivity are added to epoxy resins to create reinforced grades with enhanced electrical conductivity. Furthermore, the curing condition directly results in the degree of crosslink that is the main characteristic for controlling the final mechanical properties, flexural strength, and compressive strength, of the composites. The structure of bisphenol-A diglycidyl ether epoxy resin mainly contains epoxide groups and aromatic rings which provide high stiffness and strength. At the same time, the WCFS stack acquires a duty to absorb mechanical stress and retard the deformation of the epoxy matrix [26]. Therefore, the flexural and compressive strength of epoxy/COOH-GNP/COOH-MWCNT/WCFS composite laminates, with 1:1 of COOH-GNP:COOH-MWCNT ratio, accomplishes the DOE target (59 MPa of flexural strength and 50 MPa of compressive strength) [64]. The maximum flexural and compressive strength values are 1020 MPa and 55.9 MPa, respectively. The increases in flexural and compressive strength corresponding to the degree of crosslink were investigated to minimize the curing time (Figure 7). The curing time spent for creating the adequate quantity of crosslink bonds providing higher flexural and compressive strength than the DOE target was selected. The results of the flexural test indicate that the flexural strength escalated when the degree of crosslink increased, as shown in Figure 7a. Flexural strength or modulus of rupture represents the highest stress experienced within an epoxy composite at its moment of yield [65]. Through establishing covalent links between epoxy chains and covalent bonds between epoxy chains and the nanofillers, crosslinking the epoxy composite, which serves as a binder polymer, increases the stiffness of the linked nanofillers and WCFS and decreases their mobility. The flexural strength dramatically rose when reaching 90% of the degree of crosslink. Figure 7b shows the compressive strength of epoxy/COOH-GNP/COOH-MWCNT/WCFS composite laminates. Results exhibited that the compressive strength and the curing degree had a non-linear relationship. The compressive strength gradually increased with increasing the degree of crosslink and rising with a steep slope after 90% of the degree of crosslink. At an initial curing period of 2 min for creating 70% of the degree of crosslink, the compressive strength was unacceptable for a BP application. However, after being cured for up to 5 min with 90% of the degree of crosslink, the compressive strength progressively reached approximately 50 MPa. The experimental outcomes obviously demonstrated that the interatomic linkage between epoxide and amine groups in epoxy chains and between carboxylic groups on the filler surfaces and epoxide groups in the epoxy chains required 90 min to create 99.9% of the degree of crosslink. According to the DOE target, a 5 min curing period for creating the desired crosslink density is the threshold at which mechanical properties pass the DOE requirement.

**Figure 8:**
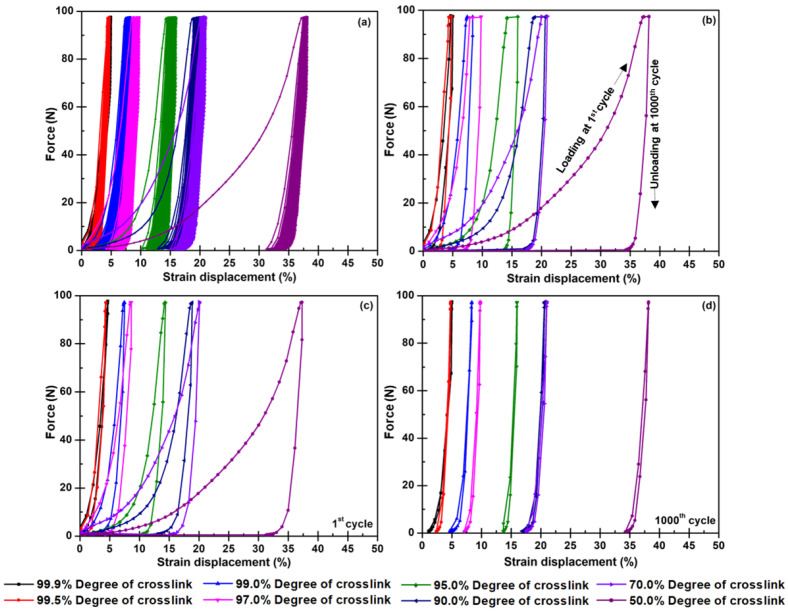
Cyclic force-strain of laminated composites with different degrees of crosslinks: (**a**) loading and unloading 1000 cycles, (**b**) loading at 1^st^ cycle and unloading at 1000^th^ cycle, (**c**) loading and unloading at 1st cycle, and (**d**) loading and unloading at 1000th cycle.

A dynamic compression test was carried out to observe the plastic strain, cyclic loading, and relaxation in long-term durability since fuel cells frequently are assembled and dismantled throughout their lifespan. Under this circumstance, there can be fatigue failures of active components such as membranes, electrodes, gas diffusion layer, and BPs [66]. Figure 8 illustrates hysteresis loops gained from 1000 loading and unloading with compressive force. The area of the hysteresis loop represents the strain displacement indicating plastic deformation as a function of time [67,68]. In terms of higher force applied in long-term durability, it may lead to cracking phenomena in a bipolar plate structure that negatively affects the machining process during the BP fabrication and gas leakage or voltage decay during the PEMFC operation. As a result, the laminated composite with full curing (>99%) provided less strain displacement than 2%. It is due to the high crosslink density created from both thermosetting and functionalized filler curing reactions. Moreover, when the composite acquires a higher degree of crosslink, the structure stabilization may be caused by the dangling end and dangling loop in its microstructure [69]. Concerning Figure 8a,b, the composites obtaining the degree of crosslink from 97% to fully curing have narrower loops than the loops of the composites with less crosslink quantity. As known, the epoxy molecules can be more flexible and can have more movement if they possess less crosslink density. Under the applied force, the molecules distribute stress by extending and rearranging. It means that the composites offering good moveability apparently give higher strain displacement. It was also found that the composite BPs with a crosslink higher than 97% degree had good shape stability during the demolding step, and provide good dimension accuracy, especially the groove configuration on BPs. Pointing to the hysteresis loop area, the loops at 1000 cycles presented the reduction of polymer chain relaxation compared to the loops at the first round. That is because of the rearrangement of the polymer chain and delocalization of nanofillers in the prepared BPs [19]. Furthermore, if loading and unloading were applied with high repetition, the WCFSs were compacted resulting in higher toughness and modulus [70]. In brief, all results convince us that the laminated composites should be cured for 10 min, which is the shortest molding time for shaping BPs with perfect dimension and flow channel configuration. 

### 3.4. Composite Structure Design to Create an Electrically Conductive Pathway

**Figure 9:**
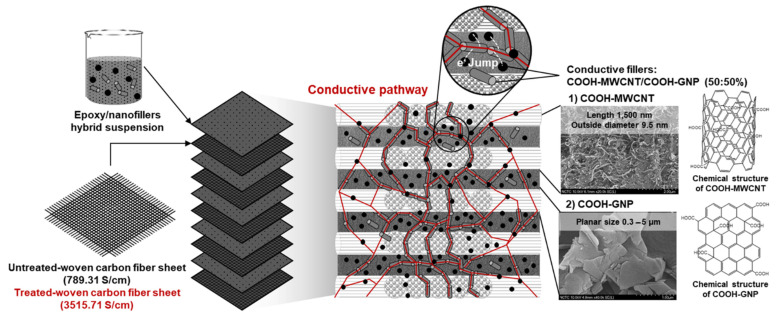
A laminated composite structure of epoxy/COOH-MWCNT/COOH-GNP/WCFS composites.

Bipolar plates are crucial for many functions inside fuel cell stacks: the collection of the generated electric current, the separation of the different cells, gas distribution over the entire electrode surface area, the eviction of the water from each cell, the humidification of the gases and cooling of the cells [13]. The desired surface and volume electrical conductivities of BPs according to the DOE requirement (75 S/cm) are required to complete the electrochemical reaction during the cell operation. The design of composite structure regarding the electrical conductivity improvement is proposed in this section. An ideal structural composite system includes continuous COOH-GNP and COOH-MWCNT particles dispersing well within the matrix, and this nanofiller localization can create an electrically conductive network with WCFS layers (Figure 9). The WCFS provides advantages in balanced in-plane mechanical properties, improved resistance to impact damage, and excellent drapeability, which is a combined effect of numerous factors such as stiffness, flexural rigidity, weight, and thickness [71,72]. The WCFS fabrication brings about complex structure features, such as curved yarn, a multitude of interfaces, resin-rich areas, and various stacking patterns [20,73]. These characteristics result in significant improvement in mechanical strength and electrical conductivity. 

**Figure 10:**
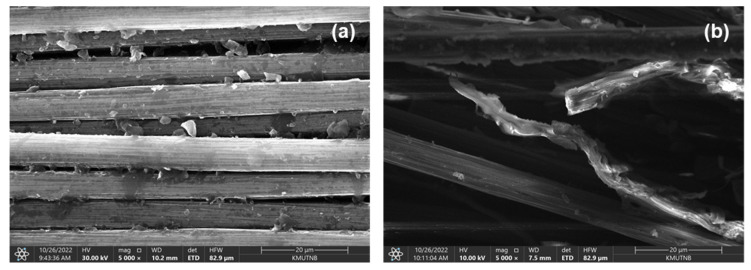
SEM micrographs of epoxy/COOH-MWCNT/COOH-GNP/WCFS composite laminates: (**a**) COOH-GNP dispersion between mat grids, and (**b**) conductive bridge connection between WCFS layers.

As a result of the incorporation of electrically conductive nanofillers into the insulating epoxy (10^−9^ S/cm of electrical conductivity), the electrical conductivity of the epoxy was increased by many orders of magnitude; 49 S/cm for epoxy/COOH-GNP composite and 62 S/cm for epoxy/COOH-MWCNT composite. The COOH-GNP and COOH-MWCNT are located at the interfaces between the WCFS layers in the composite structure. Figure 10a presents COOH-GNP distribution between the weave grids of the woven carbon fiber mat, and Figure 10b indicates that COOH-GNP disperses on the edge of the fiber mat. The COOH-MWCNT performs as a conductive bridge connecting WCFS layers. The electrical conductivity of composites including WCFS depends upon the weave pattern as well as the contacts between the adjacent tows with different directions of high-aspect-ratio fillers in a polymer matrix [32,74]. In other words, WCFS can promote the isotropic conductivity of composite BPs. In this network scenario, electrons can transpose through these conductive particles according to the percolation theory [75]. The functionalization on GNP and MWCNT surfaces significantly impacts the enhancement of the interfacial interaction between epoxy and the nanofillers towards surface roughness and active polar groups (COOH-) [30,56]. The COOH-groups result in well-interfacial interaction between COOH-nanofillers and the epoxy resin [30,48] providing an efficient conductive pathway and electron transfer.

**Figure 11:**
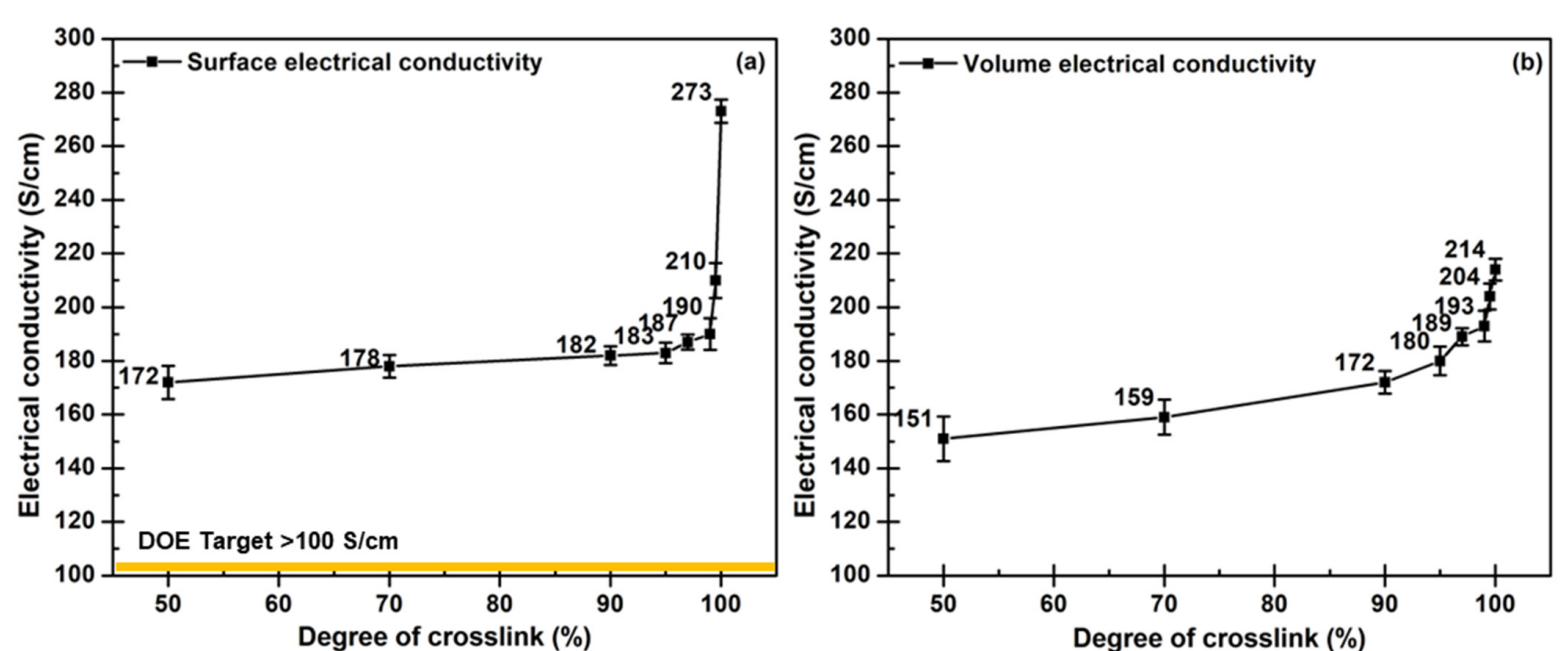
Electrical conductivity of epoxy/COOH-MWCNT/COOH-GNP/WCFS composite laminates related to the degree of crosslink: (**a**) surface conductivity, and (**b**) volume conductivity.

The results show the composite design’s success with the fillers’ synergistic effects for increasing the electrical conductivity by obtaining 214 S/cm of volume conductivity and 273 S/cm of surface conductivity. The electrical conductivity of all laminated composites is higher than 100 S/cm of the DOE target. The electrical conductivity gradually increased with the degree of crosslink increased (Figure 11). As the evidence from XPS, the functionalized nanofillers formed the covalent bonds with epoxide groups of the epoxy chains, and these bonds functioned as the crosslink between the epoxy chains. Furthermore, these bonds enhanced the performance of filler dispersion in the epoxy matrix. It is worth noting that the conductivity of the epoxy composites depends upon composite density and thickness based on various weight ratios of the epoxy composite to the WCFS layers [68,76]. The ratio also affects the viscosity of the epoxy/nanofiller suspension that is needed to achieve the desired dispersion of nanofillers on WCFS surfaces and the interface of composite laminates [28]. 

### 3.5. Bipolar Plate Performance Diagnosis

**Figure 12:**
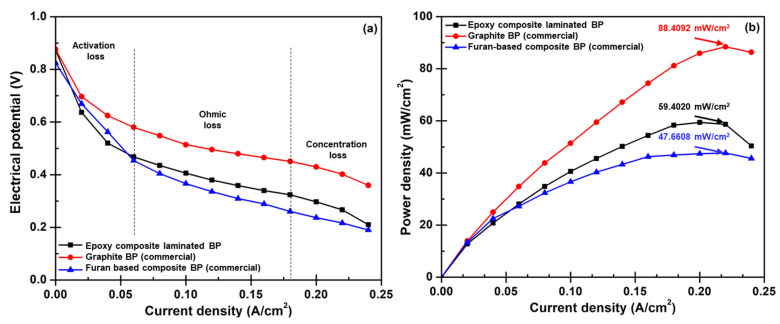
PEMFC performance tests: (**a**) polarization, and (**b**) power density.

Facilities used for a PEMFC performance test were established in the KMUTNB laboratory to validate the possibility of applying the developed BPs for the actual PEMFC operation. The BPs produced from the epoxy/COOH-GNP/COOH-MWCNT/WCFS composite laminates that acquire 97% degree of crosslink, 596 MPa of flexural strength, 52.4 MPa of compressive strength, 0.05 mm of strain displacement, 202 S/cm of surface conductivity and 193 S/cm of volume conductivity were selected for the tests under the operating condition described in the experimental section. In this activity, commercial graphite and commercial composite BPs were used for the tests as a benchmark for practicality. Results of PEMFC performance diagnoses (Figure 12) reported that the PEMFC assembled with the laminated composite BPs generated 59.4020 mW/cm^2^ of maximum power density and 71% of efficiency. However, it was still inferior to the performance of a cell that used commercial graphite BPs, particularly at the point of maximum power density. The performance of the PEMFC assembled with laminated BPs was better than the performance of the PEMFC assembled with commercial furan-based composite BPs (Table 2). The polarization curves at the middle current density region or the ohmic loss region can be interpreted that the epoxy layers increasing the internal resistance as seen from highly negative slopes of the curves gained from the furan-based composite and crated laminated composite BPs. This is an issue that needs unraveling, otherwise, the high internal and contact resistances will be the critical problem for PEMFC stack operation. Regarding BP weight lessening, this laminated composite design delivers approximately 60% weight reduction (Table 2) compared to the commercial graphite BP and 49% reduction compared to the furan-based composite. The laminated composite BP accounts for 1.1% of the total single-cell weight. This is encouraging since it means that the weight loss during the actual mobile application that is brought on by the stack weight can be significantly decreased [19]. In short, the findings demonstrate that the weight of the PEMFC stack can be decreased by using a reactive molding approach to create the laminated composite BPs.

## 4. Conclusions

Ultra-lightweight composite BPs made from epoxy/COOH-MWCNT/COOH-GNP/WCFS composite laminates were invented to deduct energy loss that is consumed for carrying the weight of the fuel cell stack. This study focuses on minimizing cycle time and determining the ideal molding temperature. In addition to understanding the reaction mechanism, the use of thermal analysis in the study of reaction kinetics is provided and explored. The need for the PEMFC application linked to mechanical qualities and electrical conductivity served as the primary indicator of the success of the production of ultra-lightweight composite BPs. The information regarding the viability of using the created BPs for the PEMFC stack in terms of energy parameters will be provided by future research. From this study, the following results were drawn:

(1) Enhancing connections between WCFS and nanofiller particles is the strategy to form a conductive network within the epoxy matrix, resulting in the enhancement of the surface and volume electrical conductivity. The WCFS layers can increase the mechanical strength of the bipolar plates, and mechanical strength is a key factor for long-term resistance to compressive force in fuel cells.
(2) The information content of thermo-analytical curves and the evaluation of kinetic parameters were found to be the most significant for the reduction of reactive molding time.
(3) Chemical bonding characterization via XPS presented the synergistic effect provided by COOH-GNP and COOH-MWCNT. These nanofillers generated the covalent bonds with epoxy molecules as crosslinking that promotes the conductive network formation and escalates the toughness of the composite BPs.
(4) The cycle time of molding epoxy composite BPs was reduced from one and a half hours to 10 min at 140 °C and under a pressure of 1500 psi.
(5) The weight of the invented composite BP accounts for 1.1% of the overall PEMFC single-cell weight, and this desired weight of BPs can reduce the overall PEMFC weight by approximately 60% in comparison with the single cell used in traditional graphite plates.
(6) The sufficed crosslinking degree for the composite BP is 97 since this degree offers the satisfied BP properties: 596 MPa of flexural strength, 52.4 MPa of compressive strength, 0.05 mm of strain displacement, 202 S/cm of surface electrical conductivity, and 193 S/cm of volume electrical conductivity. These characteristics meet the DOE requirement for PEMFC application.
(7) The efficiency of single-cell PEMFC assembled with as-fabricated composite BPs was around 71% together with 59.4020 mW/cm^2^ of maximum power density. This performance is superior to the performance of the PEMFC assembled with commercial furan-based composite BPs, however; its internal resistance must be reduced for the efficiency to be equivalent to the performance of the cell that utilized commercial graphite BPs.

This work opens up the possibility to design composite BPs based on material selection and optimized conditions of the fabrication process for high performance and long-time operation of PEMFCs.

## Figures and Tables

**Table 1:** Gaussian fitting parameter for XPS spectra of an epoxy composite.

Peak		Position (eV)	Pure Epoxy	Epoxy with COOH-MWCNT	Epoxy with COOH-GNP	Epoxy with COOH-MWCNT and COOH-GNP
C1s	C=C/C-C	285.00	35.81	34.57	31.27	29.22
	C-N	285.92	22.87	19.30	24.70	23.97
	C-O	286.74	24.18	29.33	23.21	33.66
	C=O	287.71	12.13	13.04	10.41	9.23
	O-C=O	289.23	3.08	2.51	9.35	3.23
	π-π*	291.44	1.94	1.26	1.07	0.69
O1s	O=C	529.91	32.92	19.15	38.01	20.02
	O-C	530.43	49.68	61.60	29.47	69.28
	O-C=O	531.40	17.37	13.96	24.63	8.68
	COOH	532.50	9.03	4.48	7.89	2.01
N1s	N-C	396.60	68.92	56.79	51.71	60.96
	N-O	397.37	20.44	28.41	27.87	31.94
	O-N-O	398.86	10.64	14.80	20.42	7.10

Note: The π is a bonding and π* is an anti-bonding of carbon orbital.

**Table 2:** Output data obtained from in-situ PEMFC performance tests.

Type of Bipolar Plate	OCV (V)	Efficiency at OCV (%)	Maximum Power Density (mW/cm^2^)	Weight of BP (g/Plate)	Weight of PEMFC (g/Cell)	Weight of BP Per Weight of PEMFC (%)
Epoxy composite laminated BP	0.8732	70.9	59.4020	4.832	846.504	1.1
Graphite BP (commercial)	0.8759	71.2	88.4092	12.213	861.266	2.9
Furan-based composite BP (commercial)	0.8248	67.1	47.6608	6.228	849.256	1.5

## Data Availability

The data and the code that support the results within this paper and other findings of this study are available from the corresponding author upon reasonable request.
